# Thermal conductivity of polymer composites with the geometrical characteristics of graphene nanoplatelets

**DOI:** 10.1038/srep26825

**Published:** 2016-05-25

**Authors:** Hyun Su Kim, Hyun Sung Bae, Jaesang Yu, Seong Yun Kim

**Affiliations:** 1 Mutifunctional Structural Composite Research Centre, Institute of Advanced Composite Materials, Korea Institute of Science and Technology (KIST), 92, Chudong-ro, Bongdong-eup, Wanju-gun, Jeonbuk, 55324, Republic of Korea

## Abstract

One of the most important physical factors related to the thermal conductivity of composites filled with graphene nanoplatelets (GNPs) is the dimensions of the GNPs, that is, their lateral size and thickness. In this study, we reveal the relationship between the thermal conductivity of polymer composites and the realistic size of GNP fillers within the polymer composites (measured using three-dimensional (3D) non-destructive micro X-ray CT analysis) while minimizing the effects of the physical parameters other than size. A larger lateral size and thickness of the GNPs increased the likelihood of the matrix-bonded interface being reduced, resulting in an effective improvement in the thermal conductivity and in the heat dissipation ability of the composites. The thermal conductivity was improved by up to 121% according to the filler size; the highest bulk and in-plane thermal conductivity values of the composites filled with 20 wt% GNPs were 1.8 and 7.3 W/m·K, respectively. The bulk and in-plane thermal conductivity values increased by 650 and 2,942%, respectively, when compared to the thermal conductivity values of the polymer matrix employed (0.24 W/m·K).

With the growing demand for thinner and smaller products that are integrated to ensure portability, an increasing number of studies have investigated the efficient thermal control of integrated compact-sized devices^1^,^2^,^3^,^4^. Accordingly, considerable attention is being paid to thermally conductive polymer composites that are easy to fabricate in lightweight, thin forms. Thermally conductive polymer composites are typically fabricated by introducing fillers of high thermal conductivity, such as lightweight carbonic materials^5^,^6^,^7^. Carbonic materials are characterized by particularly high values of thermal conductivity, electrical conductivity, and mechanical strength. There are various carbon allotropes with unique properties, including carbon fibres, graphite, carbon nanotubes (CNTs), graphene, and graphene nanoplatelets (GNPs)^8^.

Recently, nanocarbon materials have received particular attention because of their excellent thermal conductivity. At room temperature, thermal conduction via phonons in nanocarbons including graphenes and CNTs, is known to be theoretically limited due to Umklapp processes^9^,^10^. The thermal conductivity of single-layer graphene can be as high as 4,000–7,000 W/m·K at room temperature when measured by Raman spectroscopy and has been experimentally reported to be higher than that of CNTs^11^,^12^,^13^. GNPs vary in size and thickness depending on how they are fabricated; as a result, their reported thermal conductivity values vary considerably^2^. The large surface area of GNPs helps maximize the GNP-polymer interface, and the interface between the filler and polymer matrix has a significant impact on the thermal conductivity of thermally conductive polymer composites^14^,^15^. Weak contact at the interface produces interfacial thermal resistance and is thus an obstacle to heat flow^14^,^15^. Such interfacial thermal resistance is associated with differences in the phonon spectra, which depend on the atomic arrangements and densities of the materials^16^,^17^.

Few studies have systematically demonstrated how the thermal conductivity of composites is related to the shape, e.g., the lateral size and thickness, of the GNPs. Balandin *et al.*^18^ reported the following: 1) the GNP size and alignment in graphene laminate films were important physical parameters that affect the thermal conductivity of graphene laminate films and 2) the thermal conductivity increased linearly with the average GNP size. Shtein *et al.*^19^ reported a relationship between the thermal conductivity of polymer (epoxy) composites and the lateral size or grade of the GNP fillers. However, to illustrate a clear relationship between the thermal conductivity and lateral size or thickness of GNP fillers, more evidence (such as realistic GNP size and dispersion within polymer composites) should be provided and discussed. Three-dimensional (3D) non-destructive micro X-ray CT analysis based on X-ray sources is an appropriate analysis tool that can satisfy the requirement for extracting the realistic GNP size and dispersion within polymer composites^7^. Therefore, in this study, we investigated the relationship between the thermal conductivity of polymer (polycarbonate, PC) composites filled with five different types of GNPs (as shown in Fig. 1) and the realistic size of GNP fillers within polymer composites (as measured by the 3D micro X-ray CT analysis) while minimizing the effects of the physical parameters other than the lateral size and thickness. In addition, the thermal conductivity values of the composites were determined based on the micromechanics theory and verified by comparison with the experimental results.

## Results and Discussion

The thermal conductivity of composites containing carbon fillers is known to depend on the dispersion of the fillers, the properties of the interface between the fillers and matrix, the defect level of the fillers (which is related to their thermal conductivity), and the shapes of the fillers^14^. To investigate the relationship between the thermal conductivity of polymer composites and the lateral size or thickness of GNP fillers, the relationship should be precisely studied while minimizing the effects of the physical parameters other than the lateral size and thickness on the thermal conductivity. The dispersion of the fillers within the polymer composites is generally investigated by observing the morphology of the fracture surface of the composites using SEM. However, such an approach leads to critical distortions caused by the alteration of the internal structure of the composite samples during fracture sampling and distortion caused by two-dimensional (2D) image analysis; thus, the image data cannot be used to represent a realistic 3D dispersion of the fillers. Therefore, 3D non-destructive analysis should be applied; micro X-ray CT is one of the most useful techniques to precisely evaluate the dispersion and realistic size of fillers.

Field-emission SEM and micro X-ray CT analyses were conducted to investigate the dispersion of the fillers in the fabricated GNP/PC composites. 2D micro X-ray CT images were recorded in the direction of the sample thickness, as shown Figure S1(a); it was possible to observe the 3D internal structure of the GNP/PC composite by reconstructing the 3D micro X-ray CT images from the 2D images (see Figure S1(b)). SEM images of the fracture surfaces of the GNP/PC composites and their 3D micro X-ray CT images, are summarized in Fig. 2. The actual dispersion of the GNP fillers within the composites could not be easily evaluated using the SEM images; in contrast, the real dispersion could be clearly observed in the 3D micro X-ray CT images. Even in the composites filled with 20 wt% GNPs, the dispersion of fillers was found to be uniform regardless of the GNP type. Consequently, the effect of the dispersion of fillers on the thermal conductivity of the composites was minimized.

The surface properties of the five different types of GNPs used in this study were analysed to characterize the filler-matrix interfaces of the GNP/PC composites. Figure 3 shows the Fourier transform infrared (FT-IR) spectroscopy, X-ray photoelectron spectroscopy (XPS), and Raman scattering measurements of the GNPs with respect to their type. Figure 3(a) shows the characteristic peaks of O-H at 3421 cm^−1^ and C=O at 1654 cm^−1^, with nearly identical peak shapes, in the FT-IR spectra. The small O peak was also confirmed in the XPS results, as presented in Figs 3(b) and S2. These findings indicate that the GNPs have nearly identical surface functionalities and will exhibit similar interfacial properties as the PC matrix. Therefore, the effect of the interfacial properties on the thermal conductivity of the composites was thought to have been minimized.

Figure 3(c) shows the Raman spectra of the five types of GNPs; these results can be used as an important standard to evaluate the defect level of the fillers. In the Raman spectra, D and G bands were observed at approximately 1338 cm^−1^ and 1572 cm^−1^, respectively. The D-band is a disorder-induced feature that arises from a double resonance Raman scattering process due to non-zero-centre phonon modes, which are typically attributed to the presence of amorphous or disordered carbon atoms. The G band results from in-plane tangential stretching of the carbon-carbon bonds in the graphene sheets^20^. In other words, a lower ID/IG ratio leads to a lower filler defect level. Figure 4 show the bulk thermal conductivity of the GNP/PC composites, as defined in the figure. Based on the non-proportional relationship between the ID/IG ratio and the thermal conductivity of the composites, the defect level of the fillers was one of the minor physical parameters used to determine the thermal conductivity in the employed materials system.

Because GNPs are 2D carbon materials, there are significant differences in the thermal conductivity of the in- and through-plane directions, indicating that the thermal conductivity of composites containing GNPs could be anisotropic and that it will be necessary to measure their anisotropic (in- and through-plane) thermal conductivity values. Figure 5 shows the in- and through-plane thermal conductivity values of the GNP/PC composites, as defined in the figure. The thermal conductivity of the composites was not excellent regardless of the GNP and thermal conductivity measurement type considering the GNP thermal conductivity values of 3,000 W/m·K in the in-plane direction and 6 W/m·K in the through-plane direction^21^. These low thermal conductivity values are mainly attributed to the high interfacial thermal resistance between the GNP fillers and polymer matrix, which is known to be a physical parameter that lowers the thermal conductivity of composites by hindering phonon transport between the GNPs and polymer matrix^15^,^16^,^17^. In this study, both the bulk and in-plane thermal conductivities of the composites were enhanced with increasing GNP content. Indeed, the composites exhibited a larger increase in thermal conductivity in the in-plane direction than in the bulk direction due to the formation of thermally conductive networks inside the composites with the increasing content of 2D GNP fillers, as shown in the micro-CT images given in Figs 2 and S3.

C500 GNPs exhibited a small lateral size and thickness when compared with M5, M15, M25, and H5 GNPs and thus formed the largest interface with the PC matrix, resulting in the lowest thermal conductivity of the composites containing C500 GNPs. When M5, M15, and M25 GNP-based composites were compared in terms of thermal conductivity, a larger lateral size for the same content was associated with a higher composite thermal conductivity. Furthermore, a comparison of the thermal conductivity between M5 and H5 GNP-based composites revealed that the thermal conductivity of the composites filled with thicker GNPs was higher than that of composites filled with thinner GNPs. These findings were consistent with the theoretically calculated micromechanics results based on Mori-Tanaka theory, as shown in Fig. 4.

The relationship between the thermal conductivity of the composites and the realistic size of the GNP fillers within the polymer composites is significant because the realistic size can be altered by the applied external forces during composite fabrication. The realistic sizes of the GNP fillers within the polymer composites were measured using 3D non-destructive micro X-ray CT analysis; the statistically treated filler sizes are shown in Fig. 6. The realistic size were nearly identical to the lateral size of the raw GNP fillers, indicating that during the applied composite fabrication, excellent dispersion of the fillers was induced and the geometrical characteristics of the fillers was maintained within the fabricated composites. In conclusion, the effect of the realistic lateral size and thickness of the GNPs on the thermal conductivity of the composites was clearly revealed.

The transient temperature response behaviour of the fabricated composites is displayed in Fig. 7 to highlight the heat dissipation ability of the composites^22^. The transient temperature response of the prepared composite specimens was evaluated using an infrared camera (FLIR T420, Wilsonville, OR, USA). The composite specimens lay on an isothermal hot plate at a constant temperature of 100 °C. Thermal images of the specimens were recorded after 20 s. Subsequently, the composite specimens, at a constant temperature of 100 °C, were placed on a metal plate at room temperature. In addition, thermal images of the specimens were recorded after 20 s. The temperature increase and decrease during heating and cooling, respectively, were considerably faster in the composite with the highest thermal conductivity compared with the other composites, indicating that the high thermal conductivity provides a high heat transfer rate. Moreover, the temperature difference trend was in good accordance with the thermal conductivity trend, implying that the heat transfer rate was enhanced as a large lateral size and thick GNP fillers were introduced. Therefore, a larger the lateral size and greater thickness of the GNPs increased the likelihood that the phonon scattering at the matrix-bonded interface would be reduced, resulting in an effective improvement in the thermal conductivity and heat dissipation ability of the composite.

## Conclusion

Five types of GNPs were prepared with different lateral sizes and thicknesses and used to fabricate GNP/PC composites with different filler contents via a melt-mixing method to investigate the relationship between the lateral size and thickness of GNP fillers and the thermal conductivity of polymer composites. The realistic size and dispersion of the GNP fillers within the polymer composites were measured using 3D non-destructive micro X-ray CT analysis, and the dispersion of fillers was found to be uniform regardless of the GNP types. This indicates that the effect of the dispersion of fillers on the thermal conductivity of the composites was minimized. The findings from FT-IR and XPS revealed that the five different types of GNPs had similar surface-chemical properties; the effect of the properties of their interfaces with the PC matrix on the thermal conductivity was also minimized. The relationship between the defect level of the fillers and the thermal conductivity of the composites was not found to be proportional when assessed by Raman spectroscopy, implying that the defect level of the fillers was one of the minor physical parameters involved in determining the thermal conductivity in the employed materials system. The statistically treated size values of the GNP fillers in the composites measured using the 3D non-destructive micro X-ray CT analysis indicated the clear effect of the realistic lateral size and thickness of the GNPs on the thermal conductivity of the composites; a larger the lateral size and thickness of the GNPs increased the likelihood that the phonon scattering at the matrix-bonded interface would be reduced, resulting in an effective improvement in the thermal conductivity and heat dissipation ability of the composite.

## Experimental Method

### Materials

**Figure 1:**
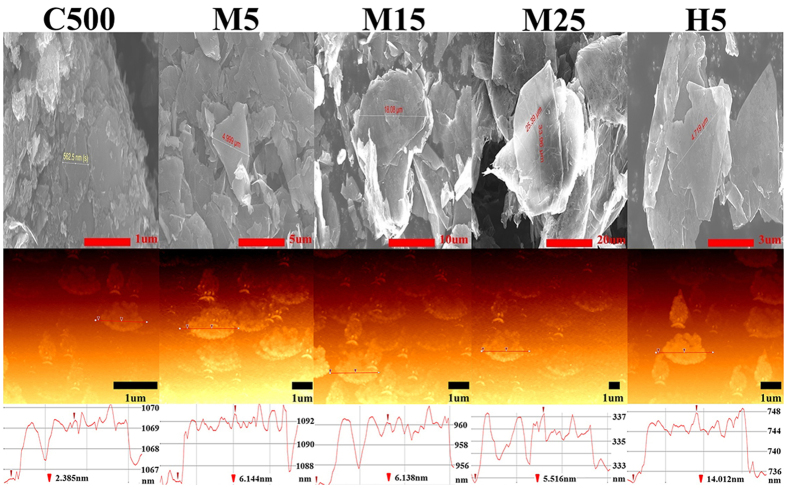
Scanning electron microscopy (SEM) and atomic force microscopy (AFM) images of GNPs showing lateral size and thickness.

**Figure 2:**
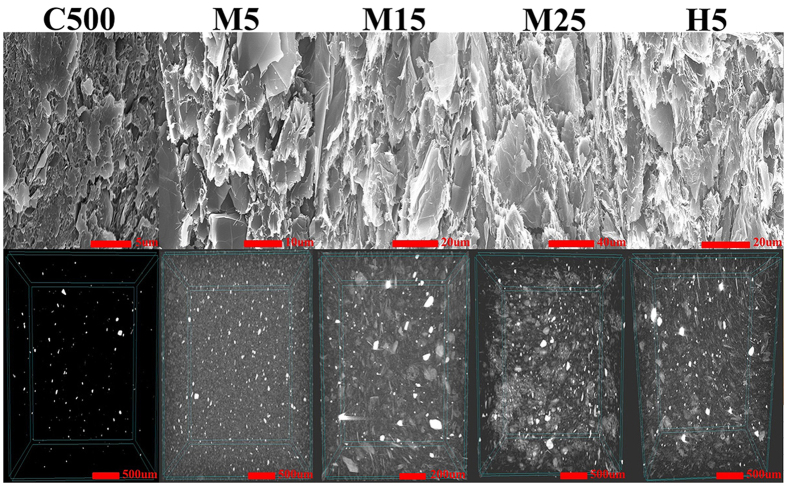
SEM and micro X-ray CT images of PC composites filled with 20 wt% GNPs of various lateral sizes and thicknesses, showing the dispersion and network of the fillers.

**Figure 3:**
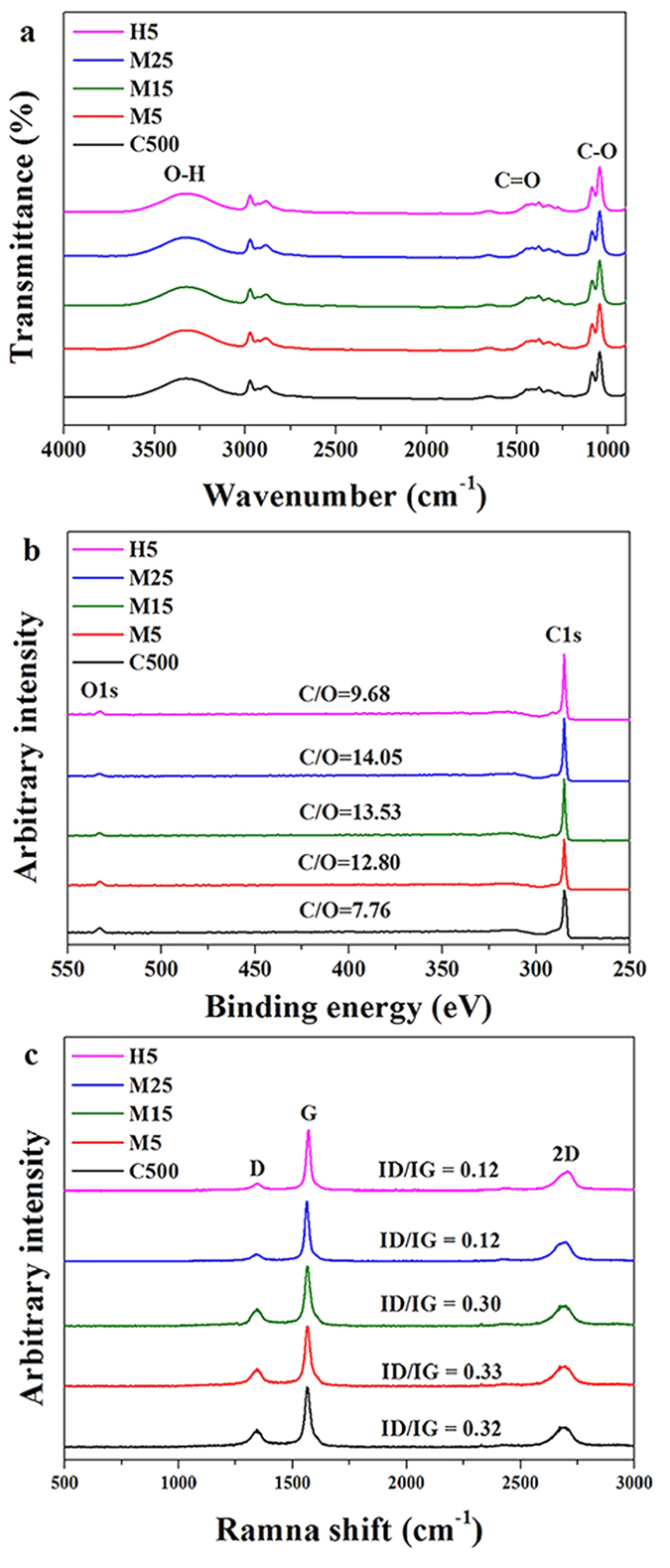
Spectroscopy results of the GNPs of various lateral sizes and thicknesses for determining their chemical and structural properties. (**a**) FT-IR spectra, (**b**) XPS spectra and (**c**) Raman spectra.

**Figure 4:**
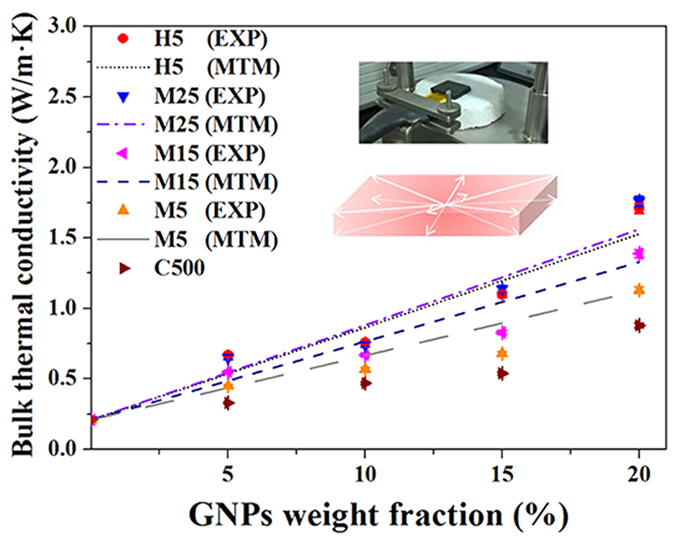
Experimentally and theoretically obtained bulk thermal conductivity vales of composites filled with GNPs of various lateral sizes and thicknesses.

**Figure 5:**
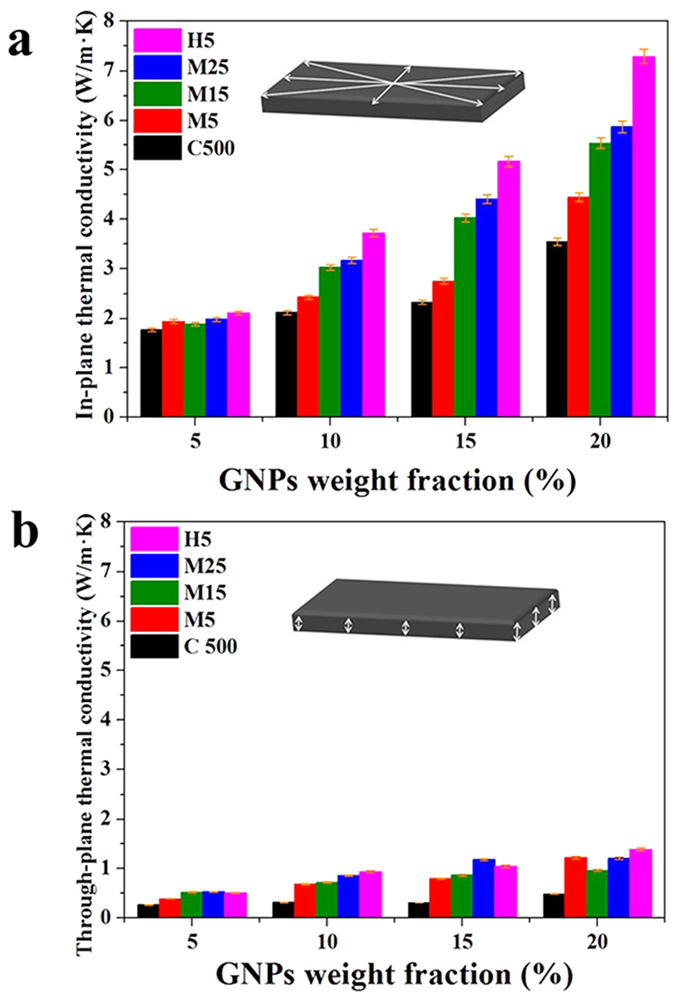
(**a**) In-plane and (**b**) through-plane thermal conductivity of composites filled with GNPs of various lateral sizes and thicknesses.

**Figure 6:**
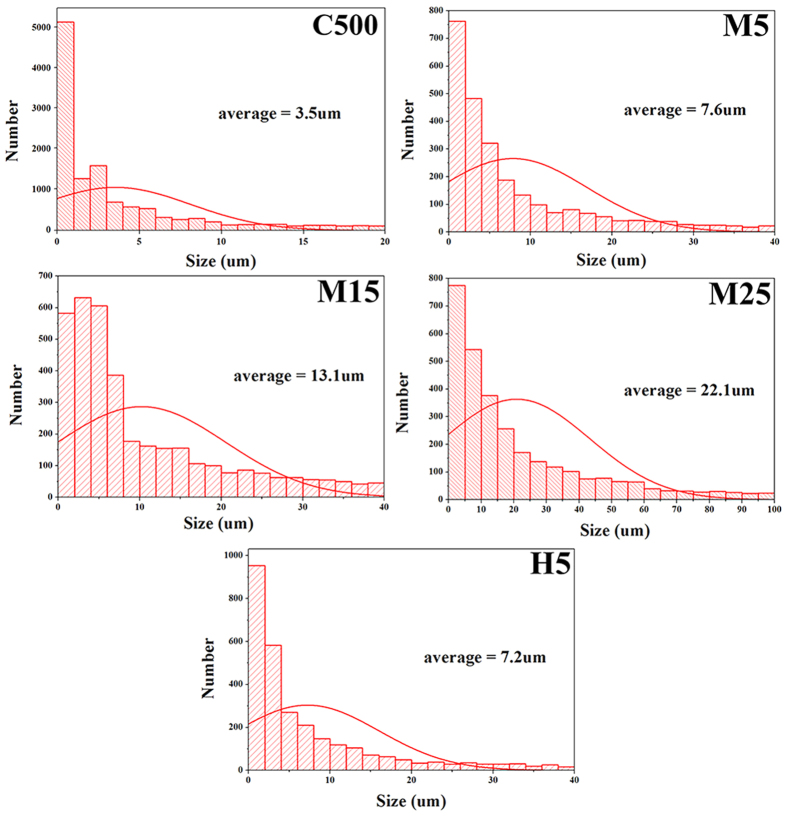
GNP size within composites statistically calculated from micro X-ray CT images.

**Figure 7:**
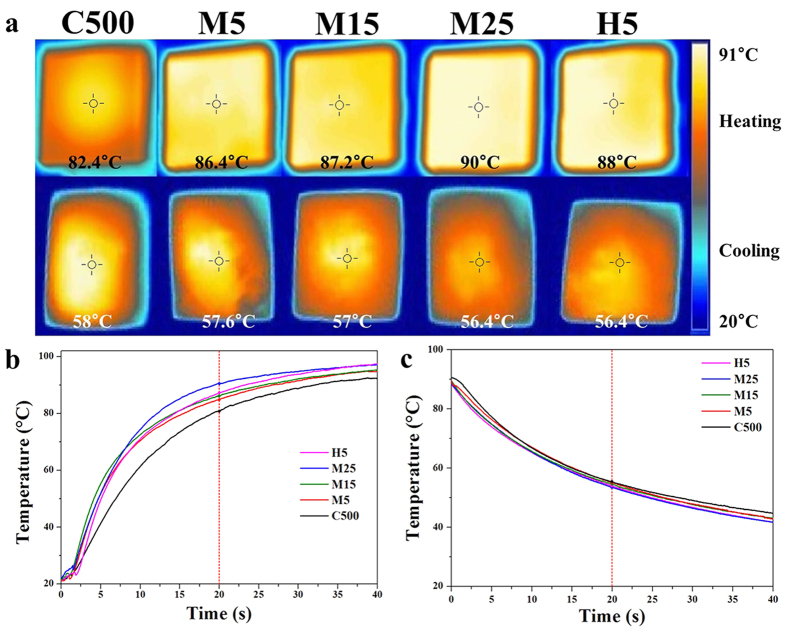
(**a**) Infrared camera images of composites during heating and cooling for transient temperature response and heat transfer measurements, and temperature-time profiles of composites during (**b**) heating and (**c**) cooling.

Five types of GNPs (C500, M5, M15, M25, and H5) were obtained from Xg Science (Lansing, MI, USA). The shapes and physical properties of the GNPs are shown in Table 1 (see Supplementary Information); the representative data are shown in Fig. 1. Details of the characterization of the fillers are shown in the Supplementary Information. A linear PC resin (LUPOY PC 1300-03, LG Chemistry Co., Gyeonggi-do, Korea) designed for compression and injection moulding was used as a matrix for the composites. The Vicat softening point of the PC was 151 °C when measured according to ASTM D 696 under 50 °C/hr and 50 N load conditions, and the resin density was 1,200 kg/m^3^ when measured according to ASTM D 792. The melt flow rate was measured as 3 g per 10 min according to ASTM D 1238.

### Preparation of composites

The GNP/PC composites were prepared using the typical melt mixing process explained thoroughly in the Supplementary Information. The characterizations of the morphology, tomography and thermal conductivity of the fabricated composites are also shown in the Supplementary Information.

## Theoretical Method

### Modified Mori-Tanaka method (MTM) for effective thermal conductivity

The MTM^23^,^24^ considers a single ellipsoidal heterogeneity embedded within an infinite homogeneous matrix domain subjected to a constant far-field heat flux, as applied to steady-state heat conduction problems. The MTM takes the opposite view and uses the continuum-averaged heat flux vector (***q***) and temperature gradient (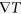) to predict the effective thermal conductivity tensor for the composite^25^,^26^. The heat flow in a composite may be characterized in terms of the far-field applied heat flux vector (***q***), i.e.,


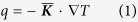


where 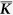 is the effective second-rank thermal conductivity tensor and 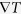 is the continuum-averaged temperature gradient. Similar to the classical Eshelby solution for linear elasticity^27^, in which the strain field inside each heterogeneity is constant, the resulting temperature gradient inside each heterogeneity is also constant when calculating the effective thermal properties. The MTM may be extended to composites containing multiple distinct heterogeneities (e.g., fibres, spheres, platelets, voids, etc.) using the multi-inclusion and multi-phase composite models^27^. Kim *et al.*^21^ used this approach to determine the elastic properties of a variety of nanocomposites. Suppose that the matrix contains *m* distinct types of ellipsoidal heterogeneities (*p* = 1, 2, …, m), each consisting of np layers (*α*_*p*_ = 1, 2, …, *n*_*p*_; *p* = 1, 2, …, m). Each type of heterogeneity has distinct thermal properties, shapes, and orientation distributions. In this case, the effective thermal conductivity tensor, 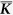, can be expressed as


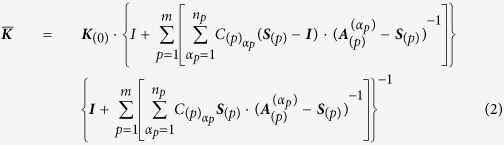


where


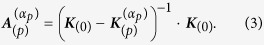


Is the second-rank temperature gradient concentration tensor for the *α*_*p*_^th^ layer of the *p*^th^ heterogeneity (*α*_p_ = 1, 2, …, *n*_*p*_; *p* = 1, 2, …, m). Here 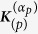 is the second-rank thermal conductivity tensor for the *α*_*p*_^th^ layer of the *p*^th^ heterogeneity, ***C***_(***p***)__***α***(*p*)_ is the volume fraction of the *α*_*p*_^th^ layer of the *p*^th^ heterogeneity, and ***S***_*(p)*_ is the second-rank Eshelby tensor common to the *p*^th^ heterogeneity and all of its layers. ***I*** is the second-rank identity tensor, and a middle dot is used to denote the tensor single dot product. The Eshelby tensor (***S***_(*1*)_) accounts for the influence of the aspect ratio/geometry of the heterogeneity on the local temperature field. In this study, the GNP aspect ratios (the ratio of diameter to thickness) were varied for the estimation. All of these parameters can be determined by experimental methods performed in the previous steps in this study via characterizations of fillers, tomography, and measurement of the thermal conductivity value of each distinct heterogeneity.

## Additional Information

**How to cite this article**: Kim, H. S. *et al.* Thermal conductivity of polymer composites with the geometrical characteristics of graphene nanoplatelets. *Sci. Rep.* **6**, 26825; doi: 10.1038/srep26825 (2016).

## Supplementary Material

Supplementary Information

## Figures and Tables

**Table 1:** Geometrical characteristics of the used GNP fillers.

	Lateral size (μm, average)	Thickness (nm, average)	Density (g/cm^3^)	Bulk density (g/cc)
C500	<2	<3	–	0.2 ~ 0.4
M5	5	6 ~ 8	2.5	0.03 ~ 0.1
M15	15	6 ~ 8	2.5	0.03 ~ 0.1
M25	25	6 ~ 8	2.5	0.03 ~ 0.1
H5	5	15	2.5	0.03 ~ 0.1
