# Supplementary material for: Thermal conductivity of polymer composites with the geometrical characteristics of graphene nanoplatelets
Source: Sci Rep. 2016 May 25;6:26825. doi: 10.1038/srep26825 (PMC4879581; doi:10.1038/srep26825)
Supplement: Supplementary Information [file srep26825-s1.pdf]

# Thermal conductivity of polymer composites with the geometrical characteristics of graphene nanoplatelets

Hyun Su Kim, Hyun sung Bae, Jaesang Yu, Seong Yun Kim*

Multifunctional Structural Composite Research Center, Institute of Advanced Composite Materials, Korea Institute of Science and Technology (KIST), 92, Chudong-ro, Bongdong-eup, Wanju-gun, Jeonbuk, 55324, Republic of Korea. * Correspondence and requests for materials should be addressed to S.Y.K. (email: sykim82@kist.re.kr)

## Experimental method

**Materials**. The characteristics of the used GNPs can be seen on the homepage of the supplier (http://xgsciences.com/products/graphene-nanoplatelets/grade-m/, /grade-c/ and /grade-h/).

**Characterization of fillers.** FT-IR (Nicolet iS 10, Thermo Fisher Scientific., USA) and XPS (AXIS-HSi, Kratos, Kyoto, Japan) were employed to observe the functionality of the GNP surface. The FT-IR spectra were measured with a resolution of 1 $cm^{-1}$ in a wavenumber range of 500-4000 $cm^{-1}$; the XPS measurements were performed using an Mg X-ray source with a power of 150 W under a pressure of 1 x $10^{-8}$ Torr. Defects in the GNPs were observed using a Raman spectroscope (LabRAM HR 800, HORIBA Jobin Yvon, Japan) with a 514-nm Ar ion laser. The Raman spectra were measured at a magnification of 50X in a wavenumber range of 500-4000 $cm^{-1}$. AFM (NX-10, Park systems Corp., Suwon, Korea) was used to observe the thickness of the GNPs. GNP samples of 0.5mg were dispersed for 2 h at 750 W in 100 ml of ethanol using an horn type ultrasonicator (VCX750, Sonics & Materials. INC., CT, USA); samples were then coated onto a silicon wafer for 60 sec at 3000 rpm using a spin coater (Spin process controller, MIDAS, Daejeon, Korea). Field emission SEM (Nova NanoSEM 450, FEI Corp., OR, USA) was used to observe the morphologies of the GNPs. Prepared samples were surface-coated with platinum for 120 sec in a vacuum using a sputter coating machine (Ion Sputter E-1030, Hitachi High Technologies, Tokyo, Japan). The coated samples were observed with a current of 10 kV applied under nitrogen vacuum conditions

**Preparation of composites.** The GNPs and the PC resin were prepared at their target contents and mixed at 250 °C with a screw speed of 60 rpm using a HAAKE Rheomix internal mixer (HAAKE™ Rheomix OS Lab Mixers, Thermo Scientific Inc., Marietta, GA, USA) for 2 h. The

composites were pelletized with filler concentrations of 5, 10, 15 and 20 wt% according to GNP types. Thermally conductive composite samples were fabricated with the pellets in a mold of 2.5 $cm^2$ with a thickness of 2 mm using a heating press (D3P-30J, Daheung Science, Incheon, Korea). The pellets were thermally compressed at a pressure of 15 MPa for 10 min at 260 °C and then quenched to 30 °C with a coolant.

**Morphology.** The fracture surface of the fabricated composites was observed using the same equipment and procedure explained previously.

**Tomography.** X-ray micro-computed tomography (Skyscan 1172, Bruker Co., USA) was used to measure the dispersion and network structures of the fillers in the composites. Measurements were made at a size of 2000 X 1332 pixels; the X-ray source was applied at a voltage of 56 kV and a current of 173 μA under normal pressure. Unfortunately, C500 GNPs cannot be observed clearly due to the resolution limitations of the micro X-ray CT.

**Thermal conductivity.** The thermal conductivity of the composites was measured under normal temperature and pressure conditions using a conductivity-measuring instrument (TPS 2500 S, Hot Disk ab, Gothenburg, Sweden), according to the ISO 22007-2 standard. We used a sensor consisting of a double spiral of thin nickel wire, which also served as a continuous plane heat source. The sensor was designed to induce a temperature rise (ΔT) by supplying a constant amount of power (P) and, at the same time, to measure temperature changes based on changes in the sensor resistance. The thermal conductivity values of the samples were determined using the Fourier equation for heat conduction based on the supplied power and the induced temperature changes.

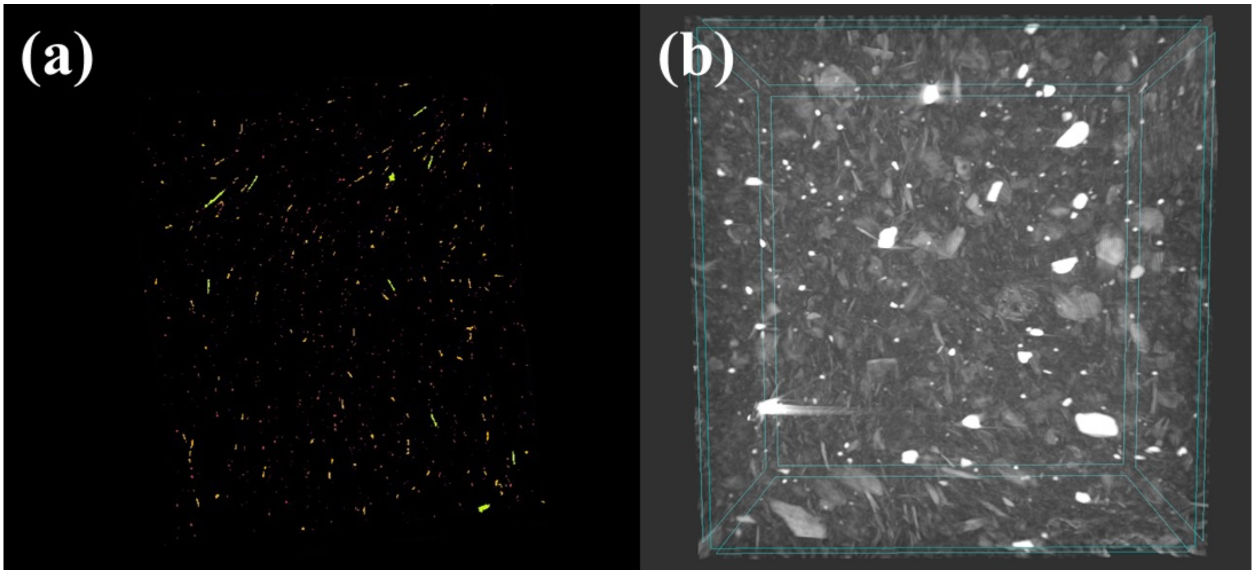

Figure S1 (a) 2D and (b) 3D Micro X-ray CT image of the composite filled with 20 wt% M15 GNP filler.

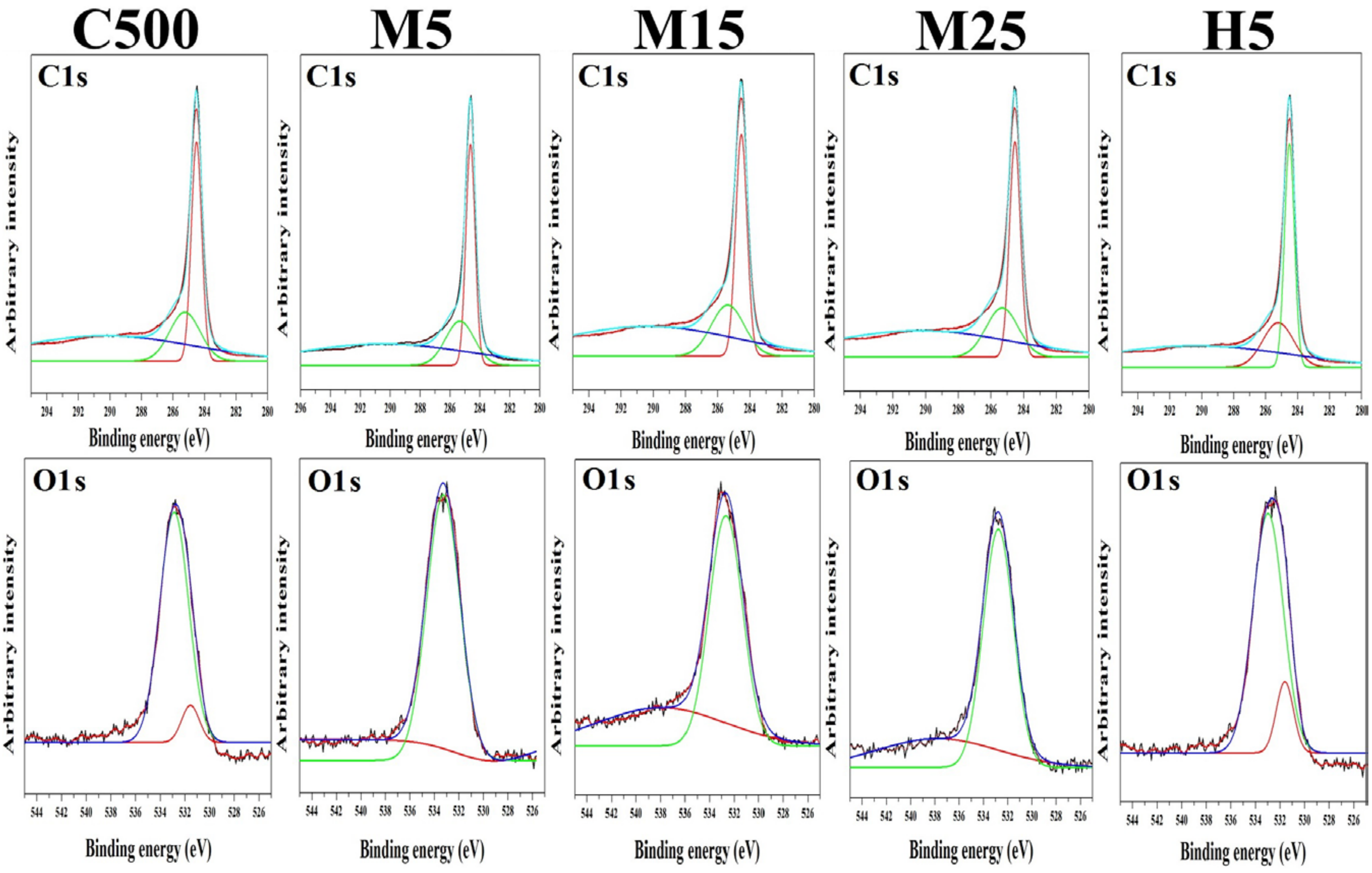


Figure S2 Chemical surface analysis based on the XPS C1s and O1s spectra of the GNP fillers.

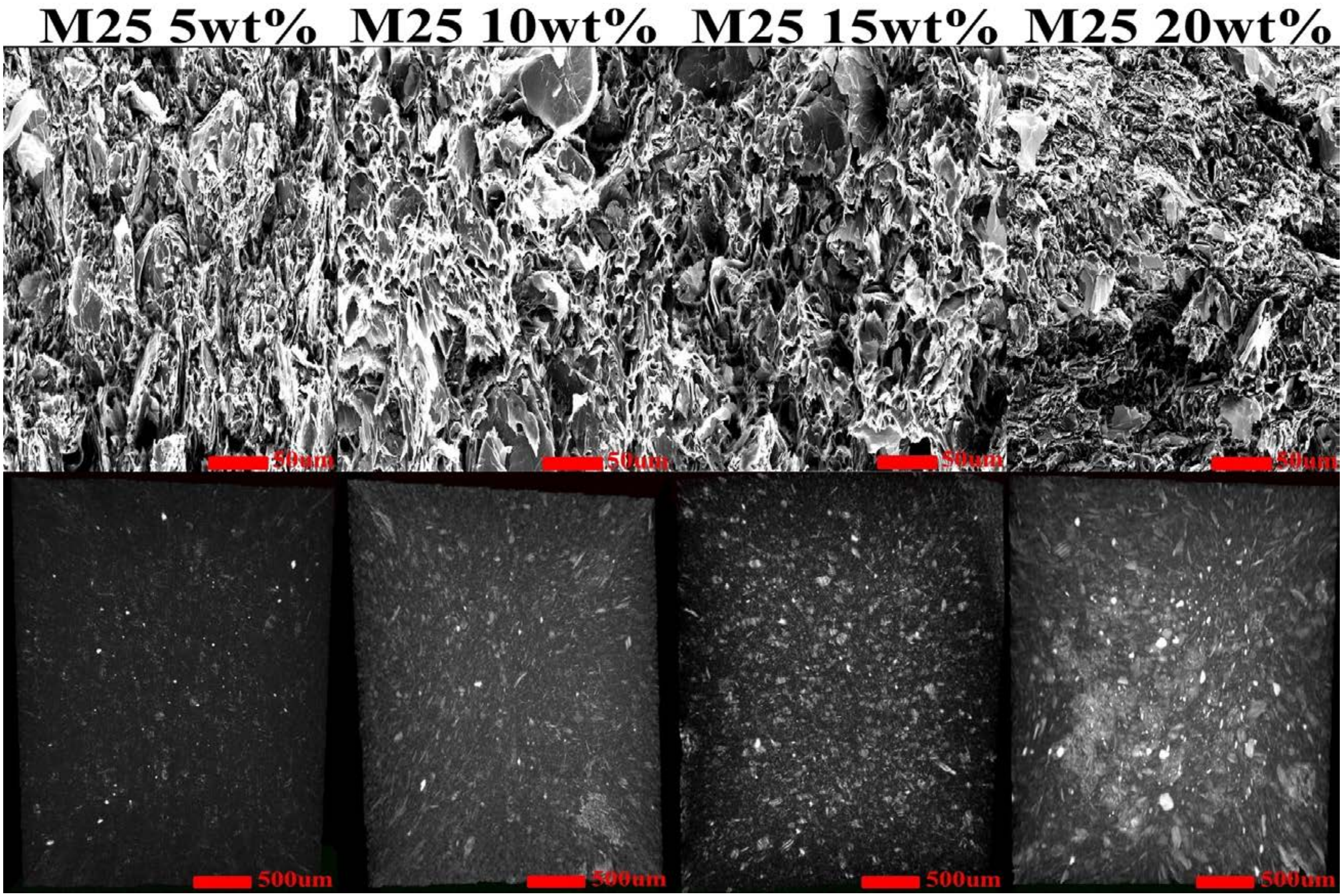


Figure S3 SEM and Micro X-ray CT images of PC composite with M25 GNPs loadings showing dispersion and network of the fillers.
